# HPV16 E7 modulates the cell surface expression of MET and CD109 via the AP2 complex

**DOI:** 10.1016/j.tvr.2024.200279

**Published:** 2024-03-12

**Authors:** Oscar Trejo-Cerro, Om Basukala, Michael P. Myers, Lawrence Banks

**Affiliations:** a International Centre for Genetic Engineering and Biotechnology, Padriciano 99, I-34149, Trieste, Italy; b Dana-Farber Cancer Institute, 450 Brookline Avenue, Mayer 440, Boston, MA, 02215, USA

**Keywords:** HPV E7, AP2 complex, CD109, MET

## Abstract

Multiple cellular pathways are affected by HPV E6 and E7 oncoproteins, including endocytic and cellular trafficking. HPV-16 E7 can target the adaptor protein (AP) complex, which contains proteins important during endocytosis transport. To further investigate the role of HPV E7 during this process, we analysed the expression of cell surface proteins in NIKS cells expressing HPV-16 E7. We show that different cell surface proteins are regulated by HPV-16 E7 via interaction with AP2. We observed that the expression of MET and CD109 membrane protein seems to be upregulated in cells expressing E7. Moreover, the interaction of MET and CD109 with AP2 proteins is disrupted by HPV-16 E7. In addition, in the absence of HPV-16 E7, there is a downregulation of the cell membrane expression of MET and CD109 in HPV-positive cell lines. These results expand our knowledge of the functions of E7 and open new potential cellular pathways affected by this oncoprotein.

## 1 Introduction

Cell trafficking mechanisms seem to be commonly targeted by different viruses to achieve a successful viral life cycle. A frequent route used for virus internalization uses clathrin-mediated endocytosis, where adaptor complexes (APs) are required to form endocytic clathrin coats [1]. Five different APs have been reported: AP1 and AP2 have a role during clathrin-coated vesicle formation, whilst AP3, AP4 and AP5 have clathrin-independent functions in protein transport [[2], [3], [4]]. Some small DNA viruses, such as adenoviruses and polyomaviruses, interact with the APs to promote their replication [5].

Human papillomaviruses (HPVs) are DNA tumour viruses that infect the basal cells of stratified epithelium. HPVs exploit the endocytosis mechanism to enter the cells; however, the specific pathway depends on the cell type and virus [6]. Interestingly, the HPV oncoproteins (E5, E6 and E7) have been associated with the regulation of endocytic trafficking, but how this contributes to the viral life cycle or during cancer development is not well understood [6]. E5 can regulate members of the epidermal growth factor receptor family (EGFRs), and it has been suggested that E5 modulates EFGR transport by controlling its trafficking from early to late endosomes via a pH-independent inhibition of vesicle fusion [7]. In addition, E5 also regulates the major histocompatibility complex (MHCI) trafficking, preventing its transport to the cell surface [8]. In the case of E6, modulation of the endosomal transport pathway through sorting nexin 27 (SNX27) has been described [9]. SNX27 protein contains a PDZ (PSD-95/DLG/ZO-1) domain that regulates the interaction with cargoes containing PDZ-binding motifs (PBMs), controlling the cellular trafficking of these proteins [10]. HPV E6 interacts with SNX27, which modulates the levels of the glucose transporter GLUT-1, affecting the glucose uptake in HPV-positive cell lines [9]. In addition, E6 can compete with TANC2 in a PBM-dependent manner to interact with SNX27 [11]. Blocking the SNX27-TANC2 interaction by E6 rescues the levels of TANC2, which promotes cell proliferation.

In the case of E7, different proteomic analyses have identified several cell trafficking-related proteins as potential E7 interacting partners; including COPA, COPE and components of the APs: AP2A1, AP2A2, AP2B1, AP2S1, AP3M1 and AP2M1 [6,[12], [13], [14]]. Interestingly, HPV-16 E7 has recently been described to contain a YxxΦ (Y, tyrosine; X, any amino acid; Φ, a hydrophobic residue) motif at its N-terminal region, which mediates the interaction with the AP2M1 subunit [13]. Furthermore, this interaction seems to be facilitated by the phosphorylation of E7 [13,14]. This AP2M1-binding motif on E7 seems to be required for an efficient E7-induced transformation. In cells expressing E7, internalization of EGFR is delayed, leading to a sustained activation of the EGFR-ERK pathway [13]. However, this phenotype is not observed in cells expressing an E7 mutant that is unable to interact with AP2M1, indicating that regulation of the endocytic transport of EGFR by HPV E7 is dependent on the interaction between this oncoprotein and the AP2 complex. All this data suggests that HPV oncoproteins can disturb the endocytic machinery and cell trafficking during the viral life cycle and carcinogenesis. In this study, we investigate whether E7 can regulate the endocytic transport of other cellular receptors through regulation of the AP2 complex.

## 2 Material and methods

### 2.1 Cell lines

SiHa and HeLa cervical cancer cell lines, as well as HEK293 cells, were maintained in Dulbecco's modified Eagle's medium (DMEM) supplemented with 10% fetal bovine serum (FBS), glutamine (300 μg/mL), and penicillin-streptomycin (100 U/mL); and incubated at 37 °C with 10% CO_2_. NIKS (normal immortal keratinocytes) cells were kindly provided by John Doorbar and were grown in F12 medium/DMEM medium (3:1 [vol/vol] F12:DMEM medium) supplemented with 10% FBS, 0.4 μg/mL hydrocortisone, 5 μg/mL insulin, 8.4 ng/mL cholera toxin, 10 ng/mL EGF, 24 ng/mL adenine, and 100 U/mL penicillin/streptomycin. The NIKS stable cell lines expressing wildtype HPV-16 E7 (16 E7Wt), 16 E7 Y25A mutant or an empty vector have been previously described [13]. Briefly, NIKS cells were transfected with the pCMV-HyPBase transposon and pB-HA/FLAG-HPV16 E7, pB-HA/FLAG-HPV16 E7 Y25A or a pPB-MCS empty plasmid (ratio of 1:2.5) and selected with puromycin (Sigma-Aldrich).

### 2.2 Plasmid constructs and transfections

The FLAG-tagged pCMV HPV-16 E7 Wt and E7 Y25A mutant have been described [13]. The EGFR-Myc/6xHis (#42665), MET-Bio/6xHis (#52022), CD109-Bio/6xHis (#51862) and AP2M1-HA (#32752) plasmids were from Addgene.

HEK293 cells were transfected by a standard calcium phosphate precipitation [15]. At 48 h post-transfection, cells were lysed and processed. For siRNA transfections, SiHa and HeLa cells were seeded at 60% confluence and transfected with siRNAs against HPV16 E6/E7 (Eurofins; a pool of three siRNAs, 5′-CACCUACAUUGCAUGAAU-3′, 5′-CAACUGAUCUCUACUGUU-3′, and 5′-CCGGACAGAGCCCAUUAC-3′), HPV18 E6/E7 (Dharmacon; 5′-CAUUUACCAGCCCGACGAG-3′), or luciferase (Dharmacon, D-002050-0-1-20), using the Lipofectamine RNAiMAX system (Invitrogen) according to the manufacturer's instructions.

### 2.3 Cell surface biotinylation

Confluent NIKS stable cell lines expressing the HPV16 E7 Wt or E7 Y25A mutant or an empty vector, or the cervical cell lines SiHa and HeLa were washed three times with ice-cold PBS and incubated with 0.5 mg of the membrane-impermeable EZ-Link Sulfo–NHS–SS-Biotin (ThermoFisher Scientific) in PBS for 30 min on ice. Free reagent was blocked with 100 mM glycine in PBS for 10 min, and cells were washed thrice with ice-cold PBS. Cells were lysed with lysis buffer (50 mM HEPES [pH 7.4], 150 mM NaCl, 1 mM MgCl2, 1% Triton X-100) supplemented with protease inhibitor cocktail I (Calbiochem) and lysates were incubated with streptavidin-conjugated Sepharose beads (GE Healthcare) for 3 h at 4 °C on a rotating wheel. The streptavidin beads were washed three times and bound proteins were sent to the Proteomic Facility at the International Centre for Genetic Engineering (Trieste, Italy) for identification by mass spectrometry; an aliquot was resuspended with Laemmli sample buffer and processed by Western blotting.

### 2.4 GST pulldown and immunoprecipitation assays

Production of the AP2 core proteins and their subsequent purification has been previously described [13]. For GST pulldown assays, HEK293 cells were transfected with the indicated constructs and, after 48 h, cells were lysed in lysis buffer supplemented with protease inhibitor cocktail I (Calbiochem). Cellular extracts were then incubated with the AP2 core purified proteins or GST-empty bound to glutathione resin (Sigma-Aldrich) for 3 h at 4 °C. After exhaustive washes, bound proteins were analysed by Western blotting.

For immunoprecipitation assays, HEK293 cells were transfected with the indicated plasmids, cells then were lysed, and cellular extracts were incubated with anti-HA beads (Sigma-Aldrich) for 3 h on a rotating wheel at 4 °C. After several washes, immunoprecipitated proteins were assessed by Western blotting.

### 2.5 Antibodies and western blotting

Mouse anti-Rb and mouse anti-AP2M1 were from BD Pharmingen; mouse anti-Memo1 and rabbit anti-AP2M1 were from Abcam; mouse anti-HPV16 E7, mouse anti-p53, mouse anti-MET, mouse anti-PTPRK, mouse anti-PTK7, mouse anti-TACSTD2, mouse anti-GAPDH and mouse anti-CD71 (transferrin receptor) antibodies were from Santa Cruz Biotechnology; rabbit anti-CD109 was from Cell Signaling Technology; mouse anti-HA-peroxidase, mouse anti-FLAG-M2-peroxidase and mouse anti-α-tubulin were from Sigma-Aldrich; mouse anti-6 × -His Tag was from Thermo Fisher Scientific.

Samples were lysed in Laemmli sample buffer and denatured by boiling for 10 min. Then, proteins were separated by SDS-PAGE and transferred to nitrocellulose membrane. Membranes were blocked with 5% non-fat dry milk in PBS-Tween (0.1% Tween 20) and incubated with primary antibodies, followed by species-specific, horseradish peroxidase-conjugated, secondary antibodies. The signal was acquired using the ECL Western blotting detection system (GE Healthcare) according to the manufacturer's instructions.

## 3 Results and discussion

**Fig. 1:**
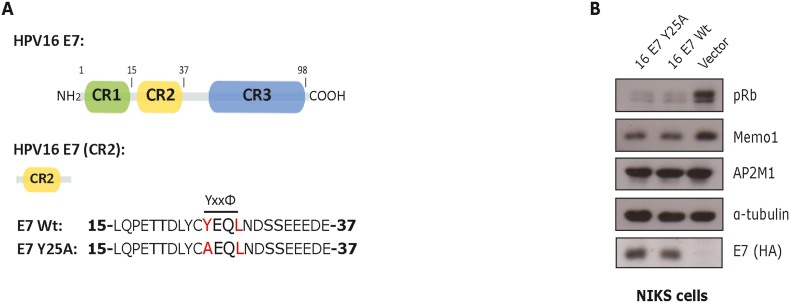
**HPV16 E7 contains a YxxΦ motif that mediates interaction with the AP2 complex.** (A) HPV16 E7 can be divided into three conserved regions (CR1-3). At the CR2 in the N-terminal region of 16 E7 Wt, there is a YxxΦ motif (Y, tyrosine; x, any amino acid; Φ, a hydrophobic amino acid). A 16 E7 mutant (Y25A) was generated. (B) NIKS stable cell lines were generated, expressing the FLAG/HA-tagged HPV16 E7 Wt, the FLAG/HA-tagged HPV16 E7 Y25A mutant or an empty vector. A representative immunoblot shows the levels of pRb, Memo1 and AP2M1. α-Tubulin was used as a loading control, and E7 expression was detected using an anti-HA antibody.

We have previously shown that HPV16 E7 contains an endocytic YxxΦ motif embedded in the CR2 region, which mediates interaction with the AP2 adaptor complex, via the AP2M1 subunit [13] (Fig. 1A). Mutation at this site (i.e., Y25A) (Fig. 1A) abolishes the interaction between E7 and AP2M1. Moreover, we observed that HPV16 E7 can regulate the endocytosis and activation of EGFR through an AP2-dependent mechanism [13]. To determine whether E7/AP2 interaction alters other endocytic transport pathways, we generated NIKS stable cell lines expressing either 16 E7 (Wt), 16 E7 Y25A mutant, or an empty vector. Cells expressing either E7 Wt or E7 Y25A mutant showed lower levels of pRb and Memo1 (Fig. 1B), which are known degradation targets of 16 E7 [[16], [17], [18]]. This data suggests that E7-AP2 complex regulation can be separated from other well-known functions of this oncoprotein.

**Fig. 2:**
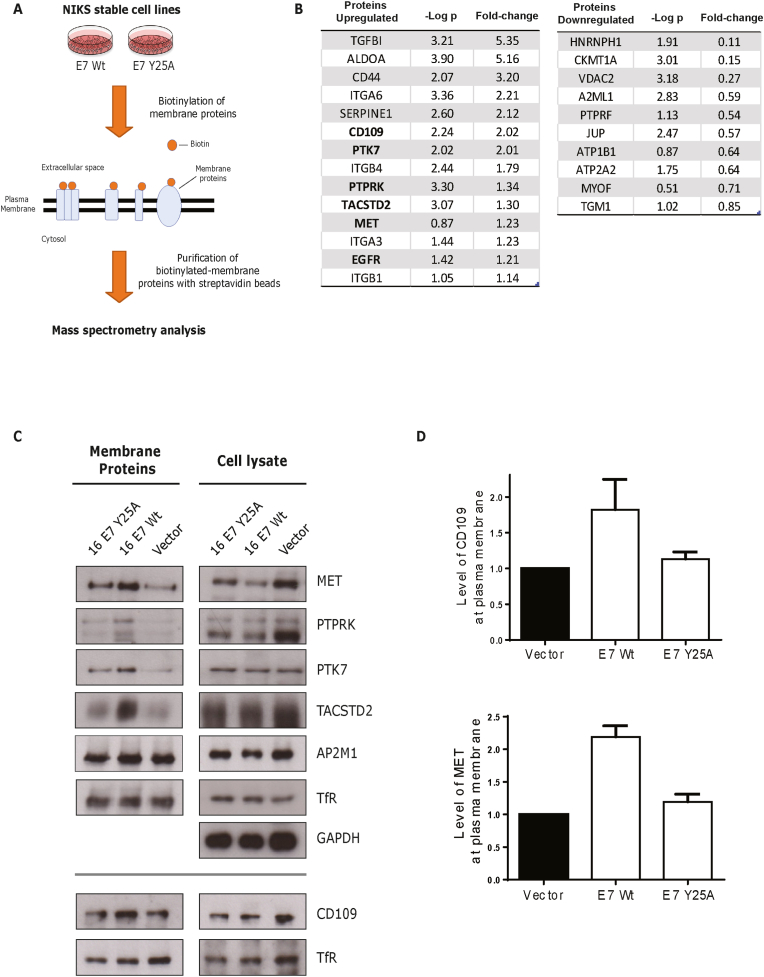
**E7 alters the cell surface proteome of NIKS cells via its YxxΦ motif.** (A) Cell surface proteins from NIKS stable cell lines expressing HPV-16 E7 Wt or E7 Y25A mutant were biotinylated with the membrane-impermeable Sulfo–NHS–SS-Biotin reagent. Cells were then lysed, and biotinylated proteins were incubated with streptavidin magnetic beads. Bound proteins were recovered, washed, and identified by mass spectrometry. (B) A list of cellular membrane proteins up- and down-regulated in NIKS-16 E7 Wt versus NIKS-16 E7 Y25A mutant is shown. The p-value (-Log p) and the Fold-change are shown. (C) Biotinylated membrane proteins from NIKS stable cell lines expressing 16 E7 Wt or E7 Y25A mutant or an empty vector were recovered using streptavidin beads. Bound proteins were lysed and analysed by Western blotting. A representative immunoblot shows the levels of CD109, MET, PTPRK, PTK7 and TACSTD2 proteins. AP2M1 and transferrin receptor (TfR) were used as loading control of membrane protein expression, and GAPDH was used as a cell lysate loading control. (D) The levels of CD109 and MET proteins at the plasma membrane were quantified from (B), where the NIKS stable cell line containing an empty vector was considered as 1.

To increase our understanding of endocytic pathways affected by E7-AP2 complex interaction, we decided to explore the cell surface proteome of NIKS cells expressing 16 E7 Wt or E7 Y25A mutant. For this purpose, cells were incubated with a membrane-impermeant biotinylating reagent, then lysed, and the biotinylated cellular proteins were identified by mass spectrometry (Fig. 2A). Remarkably, we observed several potential membrane proteins up- and down-regulated by the AP2-tyrosine binding motif in E7, including a modest upregulation of the surface EGFR (Fig. 2B), which is in agreement with our previous results [13]. In addition, we identified other tyrosine kinase receptors, such as PTK7 and MET, cell-surface glycoproteins such as CD44 and CD109 and integrin proteins such as ITGA6 and ITGB4 (full list of proteins is provided as Supplementary Fig. 1).

Next, we wanted to validate these putative cellular surface target interactions by Western blotting assays. To do this, we repeated the biotinylation of cell membrane proteins in NIKS-E7 expressing cells (Wt and Y25A mutant) or NIKS alone. Biotinylated proteins were recovered with streptavidin beads and analysed by Western blot; we observed higher total levels of the selected targets on the cell surface in Wt 16 E7-expressing NIKS cells (Fig. 2C). The receptor tyrosine kinase PTK7 is involved in cell polarity and Wnt regulation [19] and is overexpressed in patients with cervical cancer; while knocking down PTK7 expression inhibits cervical cancer cell proliferation [20]. Here, we observed that PTK7 localisation to the plasma membrane is stimulated by 16 E7. Similar results were observed with PTPRK, a protein tyrosine phosphatase identified as a tumour suppressor in different types of cancer [21]. TACSTD2 (also known as Trop2) is a cell surface glycoprotein that is frequently overexpressed in many different carcinomas [22] and, more intriguingly, its prognostic value may depend on its cellular localisation, with the membranous TACSTD2 being associated with worse survival in breast cancer [23]. Interestingly, our data indicates that TACSTD2 is stabilized at the plasma membrane in Wt 16 E7-expressing keratinocytes. Likewise, CD109, a glycosylphosphatidylinositol-anchored cell-surface glycoprotein and MET, a receptor tyrosine kinase, were also upregulated at the plasma membrane in Wt 16 E7-NIKS cells (discussed below). Moreover, 16 E7 Y25A-expressing NIKS show a localisation pattern similar to NIKS without E7, indicating that E7 can potentially regulate several cell surface proteins through AP2 complex interaction.

**Fig. 3:**
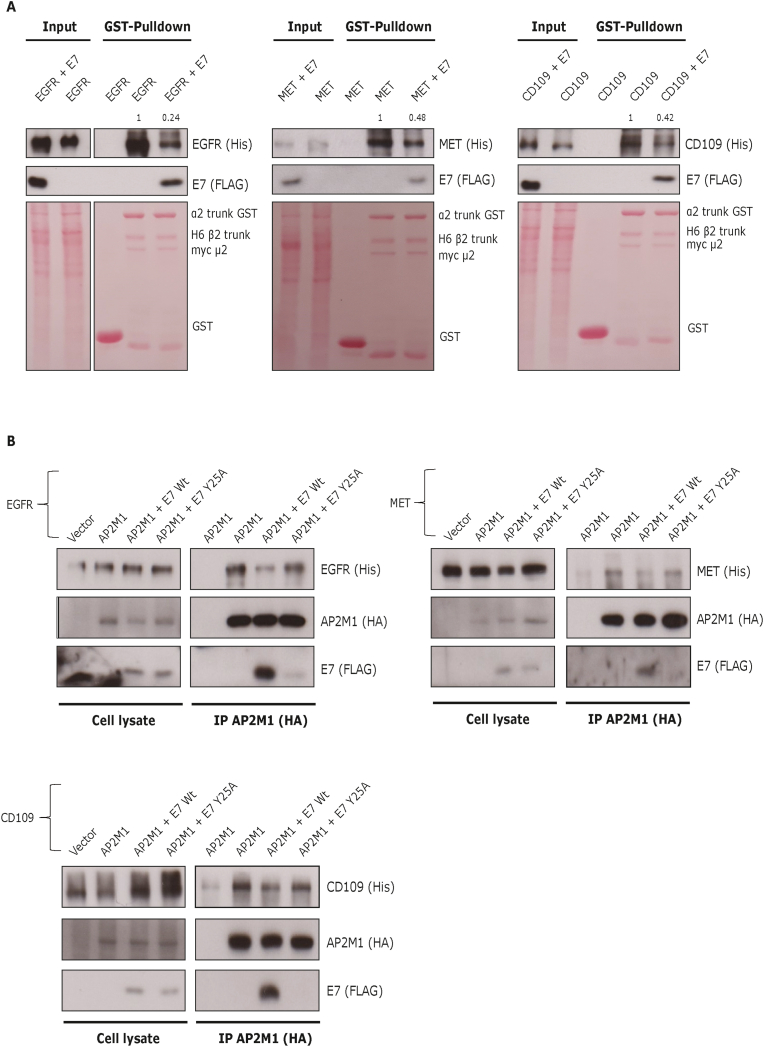
**The interaction of CD109 and MET proteins with the AP2 complex is disturbed by E7.** (A) HEK293 cells were transfected with plasmids expressing EGFR-Myc/6xHis, MET-Bio/6xHis or CD109-Bio/6xHis in the presence or absence of HPV-16 E7-FLAG/HA. After 48 h, cells were harvested and cellular extracts were incubated with the AP2 core GST (α2 trunk, β2 trunk and μ2 subunit) or empty GST, and bound proteins were resolved by Western blotting. The lower panel shows the Ponceau stain for the GST proteins. Note that less EGFR, MET and CD109 is pulled down by AP2 core in the presence of E7. The quantification of the reduction in binding is also shown above the relevant lanes on the Western blots. (B) HEK293 cells were transfected with EGFR-Myc/6xHis, MET-Bio/6xHis or CD109-Bio/6xHis, together with AP2M1-HA plasmid or an empty vector (as control) in the presence or absence of HPV-16 E7Wt-FLAG or HPV16-E7Y25A-FLAG mutant. After 48 h, cells were lysed and complexes were coimmunoprecipitated with anti-HA agarose beads and analysed by Western blotting.

EGFR internalization can be regulated by an AP2-dependent mechanism [24]. Interestingly, HPV16 E7 can occupy the cargo binding motif in AP2M1, delaying the endocytosis of EGFR and maintaining its cell surface expression [13]. The receptor tyrosine kinase MET is mostly internalised by a dynamin and clathrin-dependent mechanism [25], whilst CD109 can regulate TGF-β1 endocytosis and enhance EGFR signalling [26]. To investigate whether MET and CD109 might be potential cargoes of the AP2 complex, they were overexpressed in HEK293 cells, and cell extracts were incubated with purified GST-AP2 core proteins. As a positive control we included EGFR, previously reported as an AP2M1 interactor [13,24]. As shown in Fig. 3A, EGFR interacts with the AP2 core, as expected; similarly, MET and CD109 interact with the AP2 proteins. Both MET and CD109 contain potential endocytic YxxΦ motifs (data not shown) and MET has been shown to interact with adaptin β, a subunit of the AP2 complex [27]. Future studies will be focused on mapping the crucial motif(s) for the AP2 interaction in these cellular receptors.

More interestingly, when EFGR, MET and CD109 were overexpressed in the presence of HPV16-E7 we observed a reduction in the binding of these membrane proteins with the purified AP2 core proteins (Fig. 3A), whilst we could detect the AP2-E7 interaction, suggesting that there is competition between the cellular receptors and E7 for the binding to AP2 proteins. In the case of EGFR, E7 reduced interaction with AP2 by over 70%, whilst in the case of MET and CD109 the reduction in interaction with AP2 in the presence of E7 was around 50%. To determine whether the interaction of AP2 complex and MET and CD109 occurs *in vivo*, HEK293 cells were transfected with plasmids expressing EGFR (positive control), MET or CD109 together with HA-AP2M1; then, the cell extracts were immunoprecipitated using HA-beads. The results in Fig. 3B show that AP2M1 can interact with the indicated cellular receptors. Moreover, similar to our *in vitro* binding results, we observed a reduction in the interaction of the respective membrane proteins with AP2M1 in the presence of E7. This interaction was recovered in cells expressing the 16 E7 Y25A mutant (Fig. 3B), suggesting that E7 occupies the AP2M1 cargo binding motif, thereby preventing the interaction of the cellular receptors MET and CD109.

**Fig. 4:**
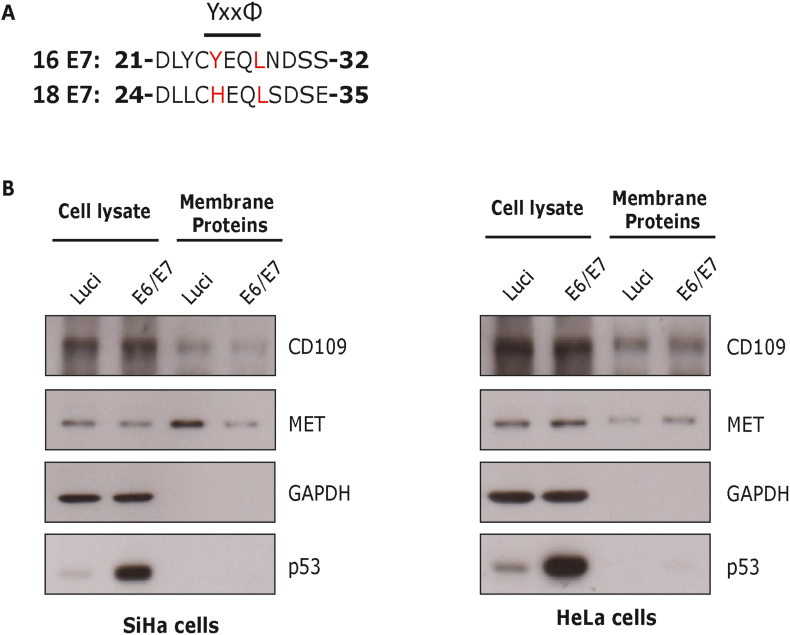
**Cell surface expression of MET and CD109 in cervical cell lines is affected by 16 E7.** (A) Protein sequence alignment of the CR2 region of HPV-16 and HPV-18 E7 proteins, encompassing the YxxΦ motif. (B) Cell surface proteins from SiHa (HPV-16-positive) and HeLa (HPV-18-positive) cells were biotinylated and purified; then cells were lysed and membrane protein expression was assessed by Western blotting.

Next, we wanted to examine whether E7 can potentially regulate the membrane proteins MET and CD109 in cells derived from cervical cancers. To investigate this, we knocked down the expression of E6 and E7 oncoproteins from HPV16 E7-expressing SiHa and HPV18 E7-expressing HeLa cells; then plasma membrane proteins were biotinylated and labelled proteins were assessed by Western blotting. Interestingly, loss of E7 in SiHa cells leads to decreased levels of MET and CD109 at the plasma membrane (Fig. 4B). However, we observed no major changes in HeLa cells upon ablation of E7, which is consistent with our previous finding that HPV18 E7 does not interact with AP2M1 [13] since it has a histidine instead of tyrosine at that position (Fig. 4A). It is worth emphasising that whilst E7 has well-recognised nuclear functions, for example association with pocket protein family members [28], there is also a substantial literature indicating cytoplasmic and membrane association of E7 [[29], [30], [31]]. Here we provide further compelling evidence of a role for these non-nuclear forms of E7.

Interestingly, MET has been recognised as an oncogene associated with Ras, PI3K and Wnt signalling pathways [32]. In addition, MET expression is higher in cervical cancer versus normal cervical tissues, and targeting MET reduces migration, invasion and proliferation in cervical cell lines [33,34]. This upregulation of MET seems to be dependent on E5 and E6 oncoproteins ([35,36], which suggests that HPV can modulate MET functions through different mechanisms. CD109 expression is also upregulated in cervical squamous cell carcinomas [37] and suppressing CD109 in cervical cell lines reduced cell proliferation and attenuated the *in vivo* tumorigenicity [38]. This phenotype was associated with the activation of the EGFR-STAT3 signalling pathway. We hypothesized that the membrane localisation of MET and CD109 is relevant for the carcinogenic phenotype in cells expressing E7. However, we cannot rule out the possibility that additional functions of CD109 are independent of this AP2-binding motif on E7. Further studies are required to elucidate which cellular pathways are affected by these cellular receptors in HPV-positive cells, and how the AP2 complex can regulate its functions. In summary, all these data together suggest that E7 can regulate several endocytic cargoes through the interaction with the AP2 complex.

## CRediT authorship contribution statement

**Oscar Trejo-Cerro:** Writing – original draft, Visualization, Validation, Methodology, Investigation, Formal analysis. **Om Basukala:** Methodology, Investigation, Formal analysis, Conceptualization, Data curation. **Michael P. Myers:** Methodology, Formal analysis, Data curation. **Lawrence Banks:** Writing – review & editing, Supervision, Resources, Funding acquisition, Conceptualization, Methodology.

## Declaration of competing interest

The authors declare that they have no known competing financial interests or personal relationships that could have appeared to influence the work reported in this paper.

The author is an Editorial Board Member/Editor-in-Chief/Associate Editor/Guest Editor for [Tumour Virus Research] and was not involved in the editorial review or the decision to publish this article.

## Data Availability

Data will be made available on request.

## References

[1] Boucrot E., Saffarian S., Zhang R., Kirchhausen T. (2010). Roles of AP-2 in clathrin-mediated endocytosis. PLoS One.

[2] Robinson M.S., Bonifacino J.S. (2001). Adaptor-related proteins. Curr. Opin. Cell Biol..

[3] Traub L.M. (2005). Common principles in clathrin-mediated sorting at the Golgi and the plasma membrane. Biochim. Biophys. Acta.

[4] Hirst J., Barlow L.D., Francisco G.C., Sahlender D.A., Seaman M.N., Dacks J.B., Robinson M.S. (2011). The fifth adaptor protein complex. PLoS Biol..

[5] Strazic Geljic I., Kucan Brlic P., Musak L., Karner D., Ambriovic-Ristov A., Jonjic S., Schu P., Rovis T.L. (2021). Viral interactions with adaptor-protein complexes: a ubiquitous trait among viral species. Int. J. Mol. Sci..

[6] Siddiqa A., Broniarczyk J., Banks L. (2018). Papillomaviruses and endocytic trafficking. Int. J. Mol. Sci..

[7] Suprynowicz F.A., Krawczyk E., Hebert J.D., Sudarshan S.R., Simic V., Kamonjoh C.M., Schlegel R. (2010). The human papillomavirus type 16 E5 oncoprotein inhibits epidermal growth factor trafficking independently of endosome acidification. J. Virol..

[8] Ashrafi G.H., Haghshenas M., Marchetti B., Campo M.S. (2006). E5 protein of human papillomavirus 16 downregulates HLA class I and interacts with the heavy chain via its first hydrophobic domain. Int. J. Cancer.

[9] Ganti K., Massimi P., Manzo-Merino J., Tomaic V., Pim D., Playford M.P., Lizano M., Roberts S., Kranjec C., Doorbar J., Banks L. (2016). Interaction of the human papillomavirus E6 oncoprotein with sorting nexin 27 modulates endocytic cargo transport pathways. PLoS Pathog..

[10] Lauffer B.E., Melero C., Temkin P., Lei C., Hong W., Kortemme T., von Zastrow M. (2010). SNX27 mediates PDZ-directed sorting from endosomes to the plasma membrane. J. Cell Biol..

[11] Broniarczyk J.K., Massimi P., Trejo-Cerro O., Myers M.P., Banks L. (2022). HPV-18E6 inhibits interactions between TANC2 and SNX27 in a PBM-dependent manner and promotes increased cell proliferation. J. Virol..

[12] Rozenblatt-Rosen O., Deo R.C., Padi M., Adelmant G., Calderwood M.A., Rolland T., Grace M., Dricot A., Askenazi M., Tavares M., Pevzner S.J., Abderazzaq F., Byrdsong D., Carvunis A.R., Chen A.A., Cheng J., Correll M., Duarte M., Fan C., Feltkamp M.C., Ficarro S.B., Franchi R., Garg B.K., Gulbahce N., Hao T., Holthaus A.M., James R., Korkhin A., Litovchick L., Mar J.C., Pak T.R., Rabello S., Rubio R., Shen Y., Singh S., Spangle J.M., Tasan M., Wanamaker S., Webber J.T., Roecklein-Canfield J., Johannsen E., Barabasi A.L., Beroukhim R., Kieff E., Cusick M.E., Hill D.E., Munger K., Marto J.A., Quackenbush J., Roth F.P., DeCaprio J.A., Vidal M. (2012). Interpreting cancer genomes using systematic host network perturbations by tumour virus proteins. Nature.

[13] Basukala O., Trejo-Cerro O., Myers M.P., Pim D., Massimi P., Thomas M., Guarnaccia C., Owen D., Banks L. (2022). HPV-16 E7 interacts with the endocytic machinery via the AP2 adaptor mu2 subunit. mBio.

[14] Trejo-Cerro O., Broniarczyk J., Kavcic N., Myers M., Banks L. (2023). Identification and characterisation of novel potential phospho-acceptor sites in HPV-16 E7. Tumour Virus Res.

[15] Chen C., Okayama H. (1987). High-efficiency transformation of mammalian cells by plasmid DNA. Mol. Cell Biol..

[16] Berezutskaya E., Yu B., Morozov A., Raychaudhuri P., Bagchi S. (1997). Differential regulation of the pocket domains of the retinoblastoma family proteins by the HPV16 E7 oncoprotein. Cell Growth Differ..

[17] Huh K., Zhou X., Hayakawa H., Cho J.Y., Libermann T.A., Jin J., Harper J.W., Munger K. (2007). Human papillomavirus type 16 E7 oncoprotein associates with the cullin 2 ubiquitin ligase complex, which contributes to degradation of the retinoblastoma tumor suppressor. J. Virol..

[18] Trejo-Cerro O., Massimi P., Broniarczyk J., Myers M., Banks L. (2022). Repression of Memo1, a novel target of human papillomavirus type 16 E7, increases cell proliferation in cervical cancer cells. J. Virol..

[19] Lu X., Borchers A.G., Jolicoeur C., Rayburn H., Baker J.C., Tessier-Lavigne M. (2004). PTK7/CCK-4 is a novel regulator of planar cell polarity in vertebrates. Nature.

[20] Sun J.J., Li H.L., Guo S.J., Ma H., Liu S.J., Liu D., Xue F.X. (2019). The increased PTK7 expression is a malignant factor in cervical cancer. Dis. Markers.

[21] Sivaganesh V., Sivaganesh V., Scanlon C., Iskander A., Maher S., Le T., Peethambaran B. (2021). Protein tyrosine phosphatases: mechanisms in cancer. Int. J. Mol. Sci..

[22] Lenart S., Lenart P., Smarda J., Remsik J., Soucek K., Benes P. (2020). Trop2: jack of all trades, master of none. Cancers.

[23] Ambrogi F., Fornili M., Boracchi P., Trerotola M., Relli V., Simeone P., La Sorda R., Lattanzio R., Querzoli P., Pedriali M., Piantelli M., Biganzoli E., Alberti S. (2014). Trop-2 is a determinant of breast cancer survival. PLoS One.

[24] Goh L.K., Huang F., Kim W., Gygi S., Sorkin A. (2010). Multiple mechanisms collectively regulate clathrin-mediated endocytosis of the epidermal growth factor receptor. J. Cell Biol..

[25] Barrow-McGee R., Kermorgant S. (2014). Met endosomal signalling: in the right place, at the right time. Int. J. Biochem. Cell Biol..

[26] Zhang J.M., Murakumo Y., Hagiwara S., Jiang P., Mii S., Kalyoncu E., Saito S., Suzuki C., Sakurai Y., Numata Y., Yamamoto T., Takahashi M. (2015). CD109 attenuates TGF-beta1 signaling and enhances EGF signaling in SK-MG-1 human glioblastoma cells. Biochem. Biophys. Res. Commun..

[27] Cho K.W., Park J.H., Park C.W., Lee D., Lee E., Kim D.J., Kim K.J., Yoon S.H., Park Y., Kim E., Cho S., Jang S., Park B.C., Chi S.W., Yoo S.H., Jang M.H., Kim H.N., Kim E., Jo K., Park Y.W. (2013). Identification of a pivotal endocytosis motif in c-Met and selective modulation of HGF-dependent aggressiveness of cancer using the 16-mer endocytic peptide. Oncogene.

[28] Dyson N., Guida P., Munger K., Harlow E. (1992). Homologous sequences in adenovirus E1A and human papillomavirus E7 proteins mediate interaction with the same set of cellular proteins. J. Virol..

[29] Smotkin D., Wettstein F. (1987). The major human papillomavirus protein in cervical cancers is a cytoplasmic phosphoprotein. J. Virol..

[30] Cesur O., Nicol C., Groves H., Mankouri J., Blair G.E., Stonehouse N.J. (2015). The subcellular localisation of the human papillomavirus (HPV) 16 E7 protein in cervical cancer cells and its perturbation by RNA aptamers. Viruses.

[31] Szalmas A., Tomaic V., Basukala O., Massimi P., Mittal S., Konya J., Banks L. (2017). The PTPN14 tumor suppressor is a degradation target of human papillomavirus E7. J. Virol..

[32] Organ S.L., Tsao M.S. (2011). An overview of the c-MET signaling pathway. Ther Adv Med Oncol.

[33] Campos-Viguri G.E., Peralta-Zaragoza O., Jimenez-Wences H., Longinos-Gonzalez A.E., Castanon-Sanchez C.A., Ramirez-Carrillo M., Camarillo C.L., Castaneda-Saucedo E., Jimenez-Lopez M.A., Martinez-Carrillo D.N., Fernandez-Tilapa G. (2020). MiR-23b-3p reduces the proliferation, migration and invasion of cervical cancer cell lines via the reduction of c-Met expression. Sci. Rep..

[34] Zhai Y., Wu W., Xi X., Yu R. (2020). Adipose-derived stem cells promote proliferation and invasion in cervical cancer by targeting the HGF/c-MET pathway. Cancer Manag. Res..

[35] Qian G., Wang D., Magliocca K.R., Hu Z., Nannapaneni S., Kim S., Chen Z., Sun S.Y., Shin D.M., Saba N.F., Chen Z.G. (2016). Human papillomavirus oncoprotein E6 upregulates c-Met through p53 downregulation. Eur. J. Cancer.

[36] Scott M.L., Coleman D.T., Kelly K.C., Carroll J.L., Woodby B., Songock W.K., Cardelli J.A., Bodily J.M. (2018). Human papillomavirus type 16 E5-mediated upregulation of Met in human keratinocytes. Virology.

[37] Zhang J.M., Hashimoto M., Kawai K., Murakumo Y., Sato T., Ichihara M., Nakamura S., Takahashi M. (2005). CD109 expression in squamous cell carcinoma of the uterine cervix. Pathol. Int..

[38] Mo X.T., Leung T.H., Tang H.W., Siu M.K., Wan P.K., Chan K.K., Cheung A.N., Ngan H.Y. (2020). CD109 mediates tumorigenicity and cancer aggressiveness via regulation of EGFR and STAT3 signalling in cervical squamous cell carcinoma. Br. J. Cancer.

